# The effect of different ammonium to nitrate ratios on antioxidant activity, morpho-physiological and phytochemical traits of Moldavian balm (*Dracocephalum moldavica*)

**DOI:** 10.1038/s41598-022-21338-6

**Published:** 2022-10-07

**Authors:** Ali Naseri, Abolfazl Alirezalu, Parviz Noruzi, Kazem Alirezalu

**Affiliations:** 1 Department of Horticultural Sciences, Faculty of Agriculture, Urmia University, Urmia, Iran; 2 Department of Food Science and Technology, Ahar Faculty of Agriculture and Natural Resources, University of Tabriz, Tabriz, Iran

**Keywords:** Biochemistry, Plant sciences, Plant physiology

## Abstract

Improving yield and secondary metabolites production of medicinal plants through nutrition management recently has been considered. The present study was done to determine the effects of different ammonium (NH_4_^+^) to nitrate (NO_3_^−^) ratios (100:0, 75:25, 50:50, 25:75, 0:100) on morphophysiological, nutrient contents (N, P, K, Ca, and Mg), phenolic compounds (Total phenolics (TPC) and flavonoid (TFC) contents and individual phenolics including chlorogenic acid, rosmarinic acid, gallic acid, cinnamic acid, caffeic acid, rutin, p-Coumaric acid, apigenin, and quercetin by HPLC–DAD), essential oil composition (by GC and GC–MS), and antioxidant capacity (by DPPH and FRAP assays) of Moldavian balm (*Dracocephalum moldavica* L.) in deep water culture (DWC) system. The highest biomass and morphological traits values of *D. moldavica* observed in 0:100 ratio of NH_4_^+^:NO_3_^−^. Also, the highest TPC and TFC was earned in plants that supplied with 0:100 ratio of NH_4_^+^:NO_3_^−^. Using the 25:75 ratio of NH_4_^+^:NO_3_^−^ caused the highest nutrient contents (N, Ca and Mg) in the leaves. p-Coumaric acid was detected as the major abundant phenolic compound in extracts and the application of 75:25 ratio of NH_4_^+^:NO_3_ resulted in the highest amounts of p-Coumaric acid, gallic acid, rosmarinic acid, caffeic acid, quercetin, and rutin. The highest antioxidant capacity by both FRAP and DPPH assays was obtained in 75:25 ratio of NH_4_^+^:NO_3_^−^. Also, the highest geranial and geranyl acetate, geraniol, and neral were obtained in 75:25, 25:75, and 50:50 ratios of NH_4_^+^:NO_3_^−^, respectively. Plants supplied with the 0:100 ratio of NH_4_^+^:NO_3_^−^, had the highest total carotenoids, while the highest chlorophyll a and b content gained with 75:25 ratio of NH_4_^+^:NO_3_^−^. These results suggest that the management of N source in nutrient recipe could contribute to enhance of morphophysiological traits, antioxidant activity and phytochemical compounds in Moldavian balm.

## Introduction

Moldavian balm or Moldavian dragonhead **(***Dracocephalum moldavica* L.**)** is an aromatic and medicinal herb that belongs to the Lamiaceae family. It is native to central Asia and is naturalized in North Africa eastern and central Europe, Northeastern United States, and China^1^. Leaves and hydro-distilled aqueous extract of Moldavian balm are used in Iranian traditional medicine especially in the West and East Azerbaijan provinces that have aphrodisiac, sedative and diaphoretic, digestive and stomachic effects. Essential oil and extracts from Moldavian balm are used widely in the food, cosmetic, flavoring and pharmaceutical industries^2^. Moldavian balm as a herbal medicine has been used for treating headaches, colds, general weakness, nerve pains, stomach and kidney spasms, and in composition of mouthwash and anti-tumor drugs^3^. Many chemical compounds have been identified in *D. moldavica* essential oil. The major of these compounds are geranial, neral, geranyl acetate and geraniol, which are oxygenated monoterpenes and are most abundant at the flowering stage^4–6^. In addition to valuable essential oil, *D. moldavica* is a rich source for antioxidants, flavonoids, phenolic acids, tannins and diterpenes^7^.

Hydroponic culture is an emerging technique that seems to be nature-friendly and can be an effective production system in countries with poor soils, land shortage and in challenge with low water resources. Therefore, hydroponics provide an excellent potential to use in that situations^8,9^. In addition to nutrient management in hydroponic systems, it is also possible to monitor and control the pH of the nutritional solution^10,11^. Deep water culture (DWC) as one of the hydroponic techniques. Since the roots are constantly supplied with oxygen-rich nutrient solution in DWC, the plants grow very quickly and vigorously. Crop production under DWC, give some advantages such as good management of pest and diseases, higher yield and notable production of secondary metabolites compared to soil culture^8,9,12^. Due to the various changes that nitrogen undergoes in the soil media, hydroponic systems are suitable to evaluate the effect of nitrogen forms in morphophysiological, growth and phytochemical traits, as well as on proteome analysis^13^. Moreover, hydroponic techniques are more suitable for assessment of root and shoot morphology and already have been used to evaluation of the effect of different nitrogen forms (NH_4_^+^ and NO_3_^−^) and their uptake efficiency in plant species^14^. In deep water culture systems, well-rooted plants are placed in a net pot on a floating plate in the liquid reservoir, like a raft, and the method is so-called as “floating culture”. To stabilize the plant, the net pot may be filled with substrate, e.g. clay balls or agricultural grade perlite. The nutrient solution reservoir, commonly have a depth at least 30 cm to allow well root growth and development. The nutrient solution regularly checked for pH and electrical conductivity to prevent nutrient imbalance. In DWC system, the plants are grown with their bare roots dipping directly into the nutrient solution which is very well oxygenated. This is done by means of an air pump and aeration stones, which introduce very fine air bubbles into the water. Considering to some plants of the same family (Lamiaceae), such as basil (*Ocimum basilicum* L.), which is grown simply in floating culture systems, good growing of Moldavian balm plant in that type of hydroponics is expected^15–17^.

Nitrogen is an essential element in plant nutrition and plays a critical role in the biosynthesis of proteins, nucleic acids and coenzymes. It is also a major component of the chlorophyll molecule and therefore plays an vital role in photosynthesis^18^. In addition, nitrogen is needed for growth, development, and productivity of higher plants. The two major forms of nitrogen normally used by higher plants are nitrate (NO_3_^−^) and ammonium (NH_4_^+^)^19^. Available forms of N has a considerable effect on growth, yield, photosynthesis, and plant quality^20,21^. In general, most plants prefer the nitrate form as a source of nitrogen, while plant roots can also absorb the ammonium form in nitrate deficiency conditions. When ammonium is provided as a main source of nitrogen with high concentrations for plants, their leaves number was decreased, growth decreased, roots stunted, and in severe condition this leads to yellowing of leaf tissue and ammonium toxicity^22^. For most species, a mixture of nitrate and ammonium is recommended as an excellent fertilizer rather than just nitrate or ammonium. The optimal ratio of nitrate to ammonium for plant growth and development depends on the genotype, environmental conditions, growth stage and the total concentration of N provided^23,24^. Increased rates of nitrogen and microelements have positive effects on leaf yield and essential oil production and the application of nitrogen and microelements can be an important role in increasing essential oil content of Moldavian balm^25^.Treating dragonhead plants with nitrogen increased plant height and branching. Fertilization with nitrogen and application of the mineral mixture caused the highest fresh herb yield and more essential oil production. Geranyl acetate, geranial and geraniol were identified as the three major compounds in the essential oil^26^. Results of a research revealed that the application of recommended rates of a NPK fertilizer in Moldavian balm growing, resulted in the highest plant height, highest number of branches per plant, highest fresh and dry weights as well as highest volatile oil and plant pigments production^27^.

Several studies have been done to evaluate the response of different species to ammonium and nitrate as a source of nitrogen. Depending on the nitrogen form supplied, species type, and environmental condition, plants show different morphophysiological and biochemical responses^28–33^. Different ratios of ammonium to nitrate in nutrient recipes has a great impact on quantitative and qualitative aspects of plants such as photosynthesis, growth and yield^34^. Increasing the ratio of ammonium to nitrate in the nutrient solution decreased most growth indices in basil (*Ocimum basilicum*)^17^. Effect of different ratios of ammonium to nitrate studied by Wang et al.^35^ Results showed that by reducing the ratio of ammonium to nitrate, the fresh and dry weight of roots and stem increased and the highest amount of the mentioned traits was gained at the 25:75 ratio of ammonium to nitrate.

Improving crop yield and enhancing secondary metabolites production of medicinal plants through nutrient minerals management recently has been considered. On the other hand, new researches have shown that the Moldavian balm, can be seen as a new unpolished diamond amongst medicinal herbs due to the variety of its possible applications in food and pharmaceutical industries. Due to the different behavior of plant species in the uptake of NO_3_^−^ and NH_4_^+^, finding the appropriate ratios of nitrate to ammonium is very important. In addition, the effect of various aspects of hydroponic culture (including the ratio of nitrate to ammonium in nutrient solution) on the production of secondary metabolites of plants is a topic that is less discussed. Due to the many uses of essential oils and other products of Moldavian balm, this plant becomes a matter of great importance in world. In the current study, in order to improve the phytochemical properties of Moldavian balm plants grown on DWC system in greenhouse, the effect of different ammonium to nitrate ratios on morphophysiological and phytochemical properties was evaluated.

## Materials and methods

### Plant materials

**Figure 1:**
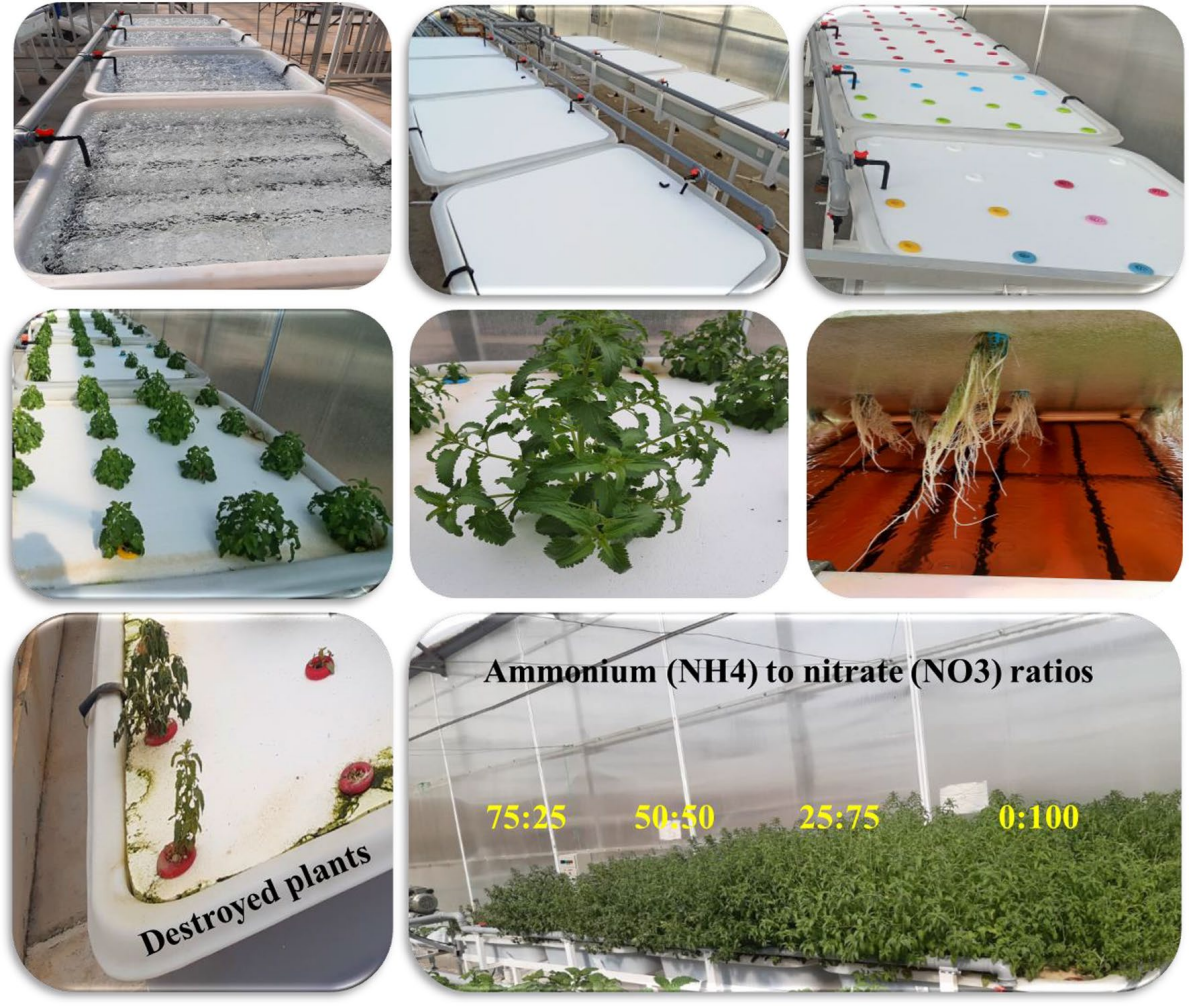
Research steps and *D. moldavica* plants with different treatments.

In order to improve the phytochemical properties of Moldavian balm (*Dracocephalum moldavica* L.) medicinal plant under greenhouse conditions and in deep water culture system, an experiment was conducted with a completely randomized design (CRD) with five treatments and three replications (five observations were used for each replication). In this experiment, the effect of different ratios of ammonium (NH_4_^+^) to nitrate (NO_3_^−^) (100:0, 75:25, 50:50, 25:75, 0:100) on the morphophysiological and phytochemical characteristics of Moldavian balm was investigated. It should be noted that plants that supplied with 100:0 ratio of NH_4_^+^:NO_3_^−^ was destroyed in mid-growth stages, due to sever ammonium toxicity. This experiment was done in the greenhouse unit and laboratories of the Urmia University in 2021. The seeds of Moldavian balm prepared from a legal local seed supplier and first sowed in 288 cells plug trays. The culture medium used for cultivation seeds was peat moss and perlite (70:30). Three weeks later, when the seedlings reached 3-4 leaves stage, they were transferred on DWC system for more growth and proceeding the main experiment. The used nutrient solutions recipe for each ratio as a treatment are shown in Table 1. In this table, the amount of all fertilizers expressed in grams. The dash signs below specific NH_4_^+^:NO_3_^−^ columns and in front of some fertilizers in Table 1, means that kind of fertilizer not used for preparing the related ratio of NH_4_^+^:NO_3_^−^. Research steps and *D. moldavica* plants with different treatments are exhibited in Fig. 1.

**Table 1:** Nutrient solution composition for two N-form treatments.

Fertilizer type*	NH_4_^+^:NO_3_¯ ratios				
	100:0	75:25	50:50	25:75	0:100
Ca (NO_3_)_2_.NH_4_.10H_2_O	–	230 g	–	600 g	930 g
KNO_3_	–	130 g	–	390 g	510 g
(NH_4_)_2_.SO_4_	990 g	742.5 g	–	247.5 g	–
NH_4_NO_3_	–	–	600 g	–	–
CaCl_2_.2H_2_O	650 g	490 g	650 g	170 g	–
KCl	372.5 g	379.3 g	372.5 g	93.125 g	–
KH_2_PO_4_	272 g	272 g	272 g	272 g	272 g
MgSO_4_	500 g	500 g	500 g	500 g	500 g

*The concentrations are for making 1000 L of standard nutrient solution with soft water.

### Morphological traits

Morphological traits were measured during full flowering stage. Plant height (cm), number of lateral branches, fresh and dry weight of aerial parts and root (g), were recorded using meter measuring tape and sensitive scale.

### Nutrient content (N, P, K, Ca and Mg)

The amount of N in Moldavian balm leaves was measured following Tedesco et al.^36^ The other elements were measured via atomic spectroscopy absorption (K, Ca, and Mg) and colorimetry (P) methods^37^. The nutrient content of Moldavian balm leaves was expressed as% in dry matter.

### Photosynthetic pigments measurement

Chlorophyll a and b (Cla and Clb) and total carotenoid content (TCC) was determined following the method of Lichtenthaler (1987) with some modification. To obtain the acetone extract, 0.5 g of dried aerial parts powder was added to 10 ml of acetone/water (80%, v/v) and ultrasound assisted extraction process was conducted (Elmasonic, Germany) at 30 °C for 35 min. The acetone extract was centrifuged at 6000 rpm for 15 min. The absorbance of the extracts was read at 645, 662 and 470 nm against the blank (80% acetone) using spectrophotometer. The amount of total chlorophyll and carotenoid content was calculated based on the following formulas:

$${\text{Chl}}_{{\text{a}}} = {12}.{\text{25A}}_{{{663}}} - {2}.{\text{79 A}}_{{{646}}} \times {\text{V}}/{\text{W}}$$

$${\text{Chl}}_{{\text{b}}} = {21}.{5}0{\text{ A}}_{{{646}}} - {5}.{\text{1 A}}_{{{663}}} \times {\text{V}}/{\text{W}}$$

$${\text{Carotenoid}} = \left( {{1}000{\text{A}}_{{{47}0}} {-}{1}.{\text{82Chl}}_{{\text{a}}} {-}{85}.0{\text{2Chl}}_{{\text{b}}} } \right)/{198} \times {\text{V}}/{\text{W}}$$

### Preparation of methanolic extract

To obtain the methanolic extract, 1 g of dried aerial parts powder was added to 10 ml of methanol/water (80%, v/v) and ultrasound assisted extraction process was conducted (Elmasonic, Germany) at 30 °C for 35 min. It was filtered using Whatman No.1 filter paper. The resulting extracts were kept at 4 °C prior to analysis^38^.

### Total phenolic content (TPC)

The Folin–Ciocalteu method was used with some modification (Slinkard and Singleton^39^). Briefly, 5 µl of extract solution was mixed with 1 ml of diluted (1:10) Folin–Ciocalteu and then, 480 µl of 7.5% sodium carbonate was added to mixture. After the solution was left for 30 min at lab temperature, the absorbance at 760 nm was measured by using a UV–vis spectrophotometer (UNICO, China). The results of total phenolic content were calculated using a standard curve provided using gallic acid (GAE) and were expressed as mg of gallic acid equivalents per gram of dry matter (mg GAE/g DW).

### Total flavonoid content (TFC)

The amount of TFC in methanolic extract of Moldavian balm was measured following Shin et al.^40^ The methanolic extract (5 μl) was added to to 200 μl of 5% NaNO_2_ and then followed after 5 min by 300 μl of 10% AlCl_3_. After further 5 min, the solution was treated with 0.2 ml of 1 mM NaOH. Finally, the solution was diluted to 1 ml with distilled water and the absorbance was recorded at 380 nm. The results of TFC were estimated using a standard curve prepared using quercetin and were expressed as mg of quercetin equivalents per gram of dry matter (mg QUE/g DW).

### Phenolic compound analysis by HPLC

The methods described by Gholizadeh-Moghadam et al.^41^ were used to quantify the phenolic compounds in alcoholic extracts using by an Agilent Technologies 1100 series HPLC (Agilent Technologies, Wilmington, DE, USA) equipped with a Dionex UltiMate 3000 system consists of a 20 μl manual sample loop, ultrasound degasser (Hwashin, Korea), quaternary pump (LPG-3400RS), column oven, and photodiode array detector with detection wavelengths of 272, 250, 310, and 360 nm (DAD-3000RS). The flow rate of 1.5 ml/min was used to separate 20 μl filtered extract (through a 0.45 μm syringe filter, Sartorius, Germany) by ZORBAX Eclipse XDB column (4.6 mm × 250 mm, 5 μm pore size, Dr. Mainsch, Germany) which was thermostatically controlled at 28 °C. Acetonitrile (solvent A) and acetic acid solution pH 3.0 (1.0% V/V in water) (solvent B) were used as mobile phases with the gradient elution program of 10%A/90%B (0–5 min), 15%A/85%B (1.5 min), 20%A/80%B (1.5 min), 25%A/75%B (1.5 min), 25-65%A/75-35%B (increase in solvent A concentration on 5% from 25-65% and decrease in solvent B concentration on 5% from 75-35 during 10-20 min), remaining on 65%A/35%B (20–25 min) and 10%A/90%B (25–35 min). Peak identification of the phenolic compounds was achieved using the retention time and photodiode array spectra comparison of commercially standard with real samples.

### Antioxidant activity

#### DPPH assay

The antioxidant activity of methanolic extract of Moldavian balm was determined using a DPPH ((2,2-diphenyl-1-picrylhydrazyl)) assay. 2000 μl of DPPH solution (0.006 gr DPPH in 150 ml methanol 80%) was added to a certain amount of extract. After 30 min of incubation at laboratory temperature, the absorbance of the solution was recorded (λ=517 nm)^42^. The inhibition% of the DPPH radical was obtained by using the following equation:

$${\text{DPPHsc \% }} = \frac{{\left( {\text{Abs control}} \right)_{{}} - { }\left( {\text{Abs sample}} \right)_{{}} }}{{\left( {\text{Abs control}} \right)_{{}} }} \times 100$$

Abs control: Absorbance of DPPH solution mixtures without extract.

Abs sample: Absorbance of DPPH mixtures containing extract.

#### Ferric reducing antioxidant power (FRAP) assay

The antioxidant activity of methanolic extract of Moldavian balm was measured by FRAP (ferric reducing antioxidant power) assay. For this, 900 μl of fresh FRAP reagent (mixing 2.5 ml of 10 mM of TPTZ solution in 40 mM HCl, 25 ml of 0.3 M acetate buffer (pH 3.6), and 20 mM FeCl_3_) was diluted with a certain amount of methanolic extract (1:10 v/v). Then, the resulting solution was heated up to 36 °C in a water bath. Finally, the absorbance of the mixture was measured at 593 nm. FeSO_4_.7H_2_O were used for the calibration curve, and shown as mM Fe^++^/g DW^43^.

### Essential oil analysis

#### Essential oil (EO) extraction.

The aerial parts of Moldavian balm were harvested during the flowering stage and dried in well ventilated shade for EO extraction. 40 g dried sample were powdered using a laboratory mill and then were subjected to hydro distillation using a Clevenger instrument. The resulting essential oil of *D. moldavica* was collected in tightly closed dark vials, and kept in darkness at 4 °C until analysis.

#### GC and GC-MS analysis

*D. moldavica* EO were analyzed by Agilent 6890 N gas chromatography (GC) system coupled with a mass spectrophotometer GC-MS-5973 with a HP-5MS non-polar capillary column (30 m × 0.25 mm × 0.250 μm) for peak separation. The oven temperature was set with heating rate of 60 to 250 °C at 5 °C/min and remaining for isothermal mode for 10 min. The injection port temperature using split mode helium gas (1:100 with a flow rate of 1 ml/min) and FID temperature were programmed at 250 °C and 280 °C, respectively. Ionization energy was 70 eV. The 1 μl sample of *D. moldavica* EO were diluted with 1:20 (v:v) n-hexane and injected through a split injector. The range of 2-800 amu was used for mass range of compounds. Retention indices (RI) were calculated by using n-alkanes (C6–C24) with the same injection conditions, and in order to identify the essential oil compounds compared with previously published data. The mass spectra of compounds were collected using X Calibur (2.07). The levels of compounds (%) were obtained according to the area normalization technique, without consideration of response factors (Davies^51^).

### Ethical approval

Authors confirm that the use of plants in the present study complies with international, national and/or institutional guidelines.

## Results and discussion

### Effects of NH4+:NO3^−^ ratios on morphological traits

**Figure 2:**
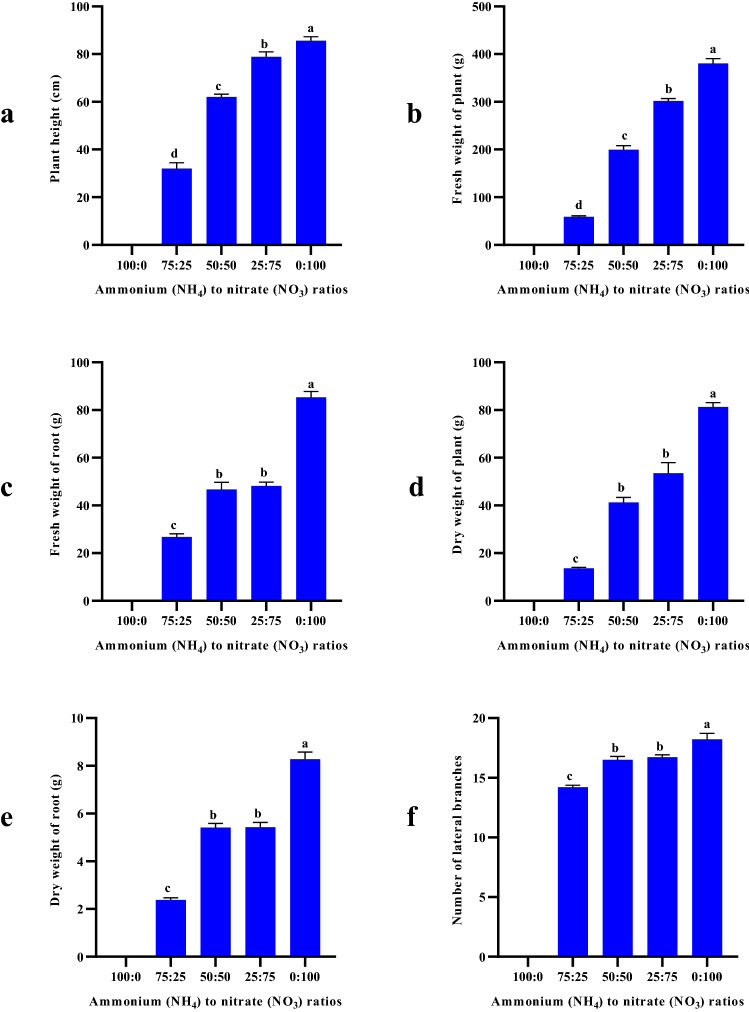
The biomass and morphological traits of *D. moldavica* in response to the different NH_4_^+^:NO_3_^−^ ratios: (**a**) plant height, (**b**) fresh weight of plant, (**c**) fresh weight of root, (**d**) dry weight of plant, (**e**) dry weight of root (**f**) number of lateral branches. *The plants of 100 NH_4_^+^:0 NO_3_^−^ treatment was destroyed in mid-growth stages, due to ammonium toxicity.

The highest biomass and morphological traits of *D. moldavica* in response to the different ratios of NH_4_^+^:NO_3_^−^, were gained at 0:100 ratio and the lowest values observed in the 75:25 ratio of NH_4_^+^:NO_3_^−^, with intermediate values obtained from 50:50 and 25:75 ratios of NH_4_^+^:NO_3_^−^ (Fig. 2). Plants that supplied with 100:0 ratio of NH_4_^+^:NO_3_^−^, was destroyed in mid-growth stages, due to ammonium toxicity. The lower dry matter content of plants in response to higher NH_4_^+^ as N source may be associated with decrease in plant height, lateral branches, fresh weight of aerial organs and roots. Improvement of growth characteristics at higher nitrate ratios has been shown in many studies. High ammonium levels in many species causes toxicity and consequently poor growth^30^. A significant increment in plant biomass was found with the increment in nitrate to ammonium ratios in *Citrullus Lanatus.* Decreasing in biomass accumulation of tomato seedlings also shown when ammonium predominated. On the other hand, NH_4_^+^ leads to a rudimentary root system development^30^. Therefore, the growth was negatively influenced by a high levels of NH_4_^+^ in the solution^29^. Nitrogen fertilizer is one of most important factors affecting in *Ocimum basilicum* production. In *O. basilicum*, the supply of ammonium (NH_4_^+^), even in the presence of nitrate (NO_3_^−^), significantly impaired plant growth (Biesiada & Kuś, 2010; Kiferle et al.^17^). There are various reasons and mechanisms for ammonium toxicity in different species including disturbance in acid–base balance, reduced protein glycosylation, acidification of the external environment, and the energy lost exporting excess ammonium^44^. In addition, root growth in plant species is sensitive to excess NH_4_^+^^45^ and inhibition of root development may related to ethylene signaling^46^ or auxin transport^30,47^. Photosynthesis is perhaps the most important chemical process in plant growth. Species supplied with ammonium as main N source are suffered by decreased rates of net photosynthesis^30,48^. The negative effect of NH_4_^+^ on net photosynthesis rate has been attributed to excessive concentration of NH_4_^+^ in leaf tissue, which can cause separation of the electron transport reactions from phosphorylation in chloroplasts^49^.

### Nutrient content (N, P, K, Ca and Mg)

Though, the beneficial components of medicinal plants that possess therapeutic values are mostly classified to tannins, alkaloids, steroids, polyphenolic acids and etc., the macro and micro nutrients are generally essential for metabolites production in the medicinal plants^50^. The macronutrients are consumed in larger quantities and are present in plant tissues^51,52^. The different NH_4_^+^:NO_3_^−^ ratios in the hydroponic nutrient solution significantly (*p* < 0.01) changed the N, P, K, Ca and Mg content in the leaf tissue. The highest and lowest leaf N, Ca and Mg contents of *D. moldavica* were observed in 25:75 and 75:25 ratio of NH_4_^+^:NO_3_^−^, respectively. Low NH_4_^+^:NO_3_^−^ ratios in the hydroponic nutrient solution generally resulted in greater leaf N, Ca and Mg content (Table 2). Also, Leaf P and K contents of *D. moldavica* were the highest with 75:25 ratio of NH_4_^+^:NO_3_^−^, whereas the lowest values obtained with 0:100 and 50:50 ratios of NH_4_^+^:NO_3_^−^ (Table 2).

**Table 2:** The nutrient content of *D. moldavica* in response to the different NH_4_^+^:NO_3_^−^ ratios.

NH_4_^+^:NO_3_^−^ ratios	Nutrient content (%)					
	DM	N	P	K	Ca	Mg
100:0*	–	–	–	–	–	–
75:25	97.80 ± 0.50a	2.00 ± 0.11d	1.69 ± 0.03a	4.00 ± 0.14a	1.04 ± 0.02c	0.84 ± 0.01c
50:50	97.70 ± 0.40a	2.52 ± 0.26c	0.79 ± 0.03b	3.57 ± 0.06c	1.56 ± 0.08a	1.20 ± 0.02b
25:75	97.50 ± 0.40a	4.06 ± 0.15a	0.71 ± 0.01c	3.65 ± 0.09bc	1.64 ± 0.07a	1.39 ± 0.07a
0:100	96.90 ± 0.30a	3.01 ± 0.09b	0.52 ± 0.02d	3.79 ± 0.10b	1.40 ± 0.02b	1.27 ± 0.08b

*The plants of 100 NH_4_^+^:0 NO_3_^−^ treatment was destroyed in mid-growth stages, due to ammonium toxicity.

*DM* Dry matter.

Prior researches has reported that plant species fed with ammonium as the main N source, contain higher concentrations of anions such as P and lower concentrations of cations such as Ca and Mg than those species fed with nitrate as the main N source^28,53^. The likely reason for the high concentration of K in plants grown in high ammonium is that plants use K accumulation as an alleviative mechanism to NH4^+^ toxicity^54^. Plants fed with ammonium, generally contain less low-molecular weight anions (nitrate) and thus have less negative charges to balance (Zhang et al.^55^). Phosphorus have a critical role in in providing anion and charge balancing the ammonium-fed plant. Therefore, high levels of phosphorus in ammonium-fed nightshade plants may be participate in positive and negative charges balancing in the plant. Generally, high levels of some nutrients such as N and P were observed in plants fed with mixture of ammonium and nitrate as N source^30^. Compared to the sole NO_3_^−^ as N source (0:100 ratio of NH_4_^+^NO_3_^−^), the supply of NH_4_^+^ affect the balance of N, P, and K in plant organs (Zhang et al.^32^). A decrease in Mg and K contents was observed in *Eustoma grandiflorum* plants fed with the sole NH_4_^+^ as N source^56^.

### Photosynthetic pigments

Chlorophyll plays a vital role in the photosynthetic processes in plants. It is not only responsible for capturing solar energy to carry out photosynthesis, but also plays a role in photoprotective processes and shows antioxidant activity, all of which contribute to effective biomass and oxygen production^57–59^.

**Figure 3:**
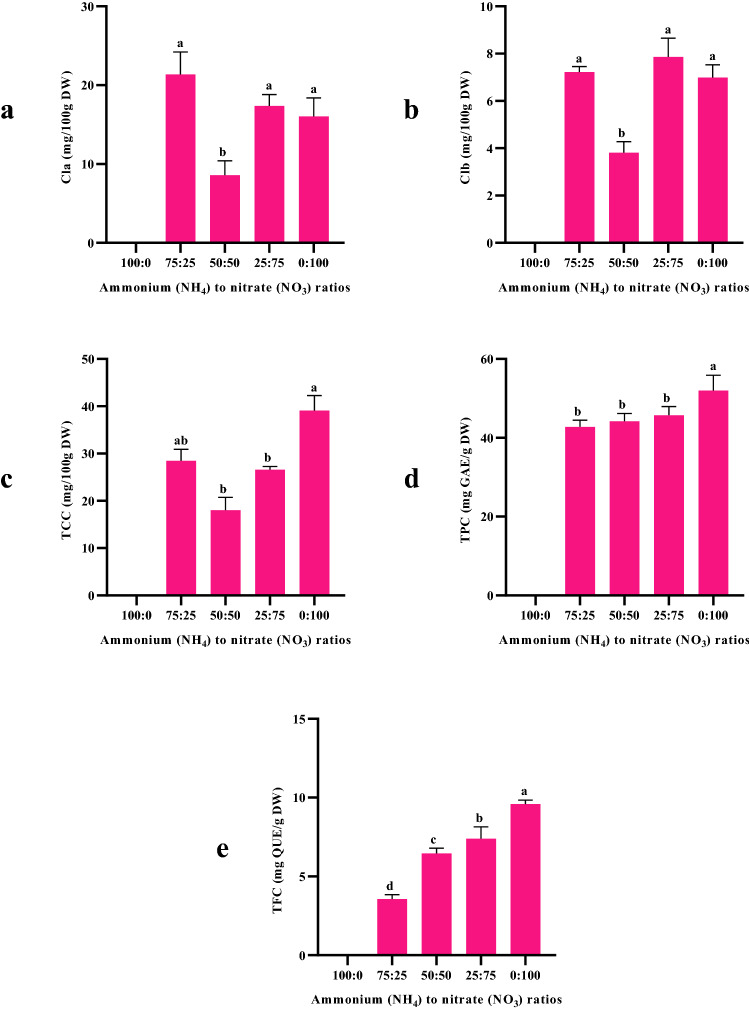
The phytochemical content of *D. moldavica* in response to the different NH_4_^+^:NO_3_^−^ ratios: (**a**) chlorophyll a (Cla) (**b**) chlorophyll b (Clb) (**c**) total carotenoid content (TCC) d) total phenolic content (TPC) e) total flavonoid content (TFC). *The plants of 100 NH_4_^+^:0 NO_3_¯ treatment was destroyed in mid-growth stages, due to ammonium toxicity.

The amounts of chlorophyll a, chlorophyll b, and total carotenoid content of aerial parts in the different treatments are exhibited in Fig. 3a–c. Moldavian balm plants fertilized with the 0:100 ratio of NH_4_^+^:NO_3_^−^, had the highest total carotenoid content, while the highest and lowest chlorophyll a and b contents were observed in 75:25 and 50:50 ratios of NH_4_^+^:NO_3_^−^, respectively. There was no significant difference between other treatments. Nitrogen is an essential nutrient in plant cells found in many (macro) molecules such as proteins, chlorophyll, pigments, nucleic acids and secondary metabolites^60,61^. Different nitrogen forms have a various effect on the amount of photosynthetic rate and pigments of higher plants (Xiao et al.^62^). In the present study, higher NO_3_^−^ and NH_4_^+^ levels both increased photosynthetic pigments (chlorophyll a, b and carotenoid) content. These results are in conflict with some findings mentioned in other scientific papers and this may be due to unrevealed matters in uptake of both forms of N. Several previous studies reported that using NO_3_^−^ as main N source, increases the availability of nitrogen and magnesium. These elements are a part of photosynthetic pigments and increases their content. Similar results were found by Liu et al.^29^ and Qadir et al.^63^.

### Effect of the NH4^+^:NO3^−^ ratios on TPC and TFC

Phenolic compounds as well as flavonoids are important bioactive agents that have long been interested due to their benefits for human health^64–67^. The results showed that TPC and TFC in *D. moldavica* were affected by various ratios of NH_4_^+^:NO_3_^−^ (*p* < 0.01). The highest and lowest TPC and TFC in *D. moldavica* were obtained in 0:100 and 75:25 ratios of NH_4_^+^:NO_3_^−^, respectively, which are shown in Fig. 3d,e. Moldavian balm plants treated by nutrient solution with high concentrations of NO_3_^−^, produced greater amounts of these compounds, significantly. The result of present study is in agreement with some previous works and supports their results^32,33^. Decreasing of phenolics content in plant tissues (leaves and roots) due to the supply of NH_4_^+^ as sole N source reported in several plant species such as pea^68^, corn^69^, and *Echinacea angustifolia*^70^. Ammonium may by altering intracellular acidity affect the biosynthesis of metabolites including phenylpropanoid pathway^71^. There is less information on how the N source affects the biosynthesis and accumulation of various secondary metabolites including phenolics and most mechanisms involved are still unclear^72^.

### Phenolic compound analysis by HPLC-DAD

**Figure 4:**
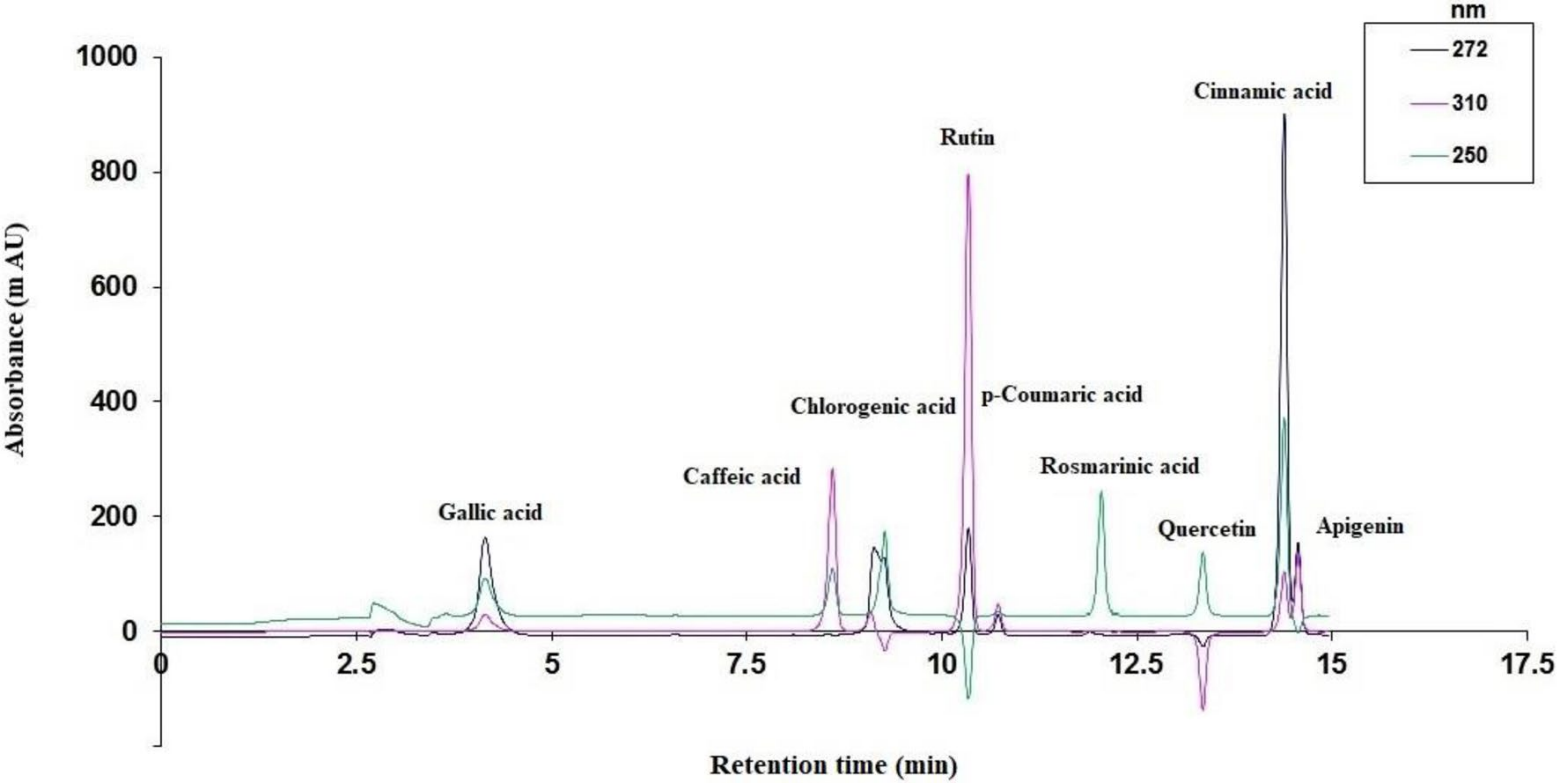
The chromatograms of phenolic acids and flavonoids standards.

Table 3 shows the retention time of the phenolic compounds studied, calibration curve, the correlation coefficient and the limits of detection (LOD) and quantification (LOQ). The chromatograms of phenolic acids and flavonoids standards are exhibited in Fig. 4. Phenolics as antioxidant are important due to their therapeutic potential related with many diseases such as anti-inflammatory, Alzheimer's and anti-cancer^73^. The contents of individual phenolics (gallic acid, rosmarinic acid, chlorogenic acid, caffeic acid, cinnamic acid, p-Coumaric acid, apigenin, quercetin, and rutin) by HPLC-DAD in the different treatments of NH_4_^+^:NO_3_^−^ are exhibited in Table 4. The results revealed that phenolic composition in methanolic extracts of *D. moldavica* were considerably influenced by the various ratios of NH_4_^+^:NO_3_^−^ (*p* < 0.01). p-Coumaric acid was identified as the most abundant compound in extracts. The highest amounts of p-Coumaric acid, gallic acid, rosmarinic acid, caffeic acid, quercetin, and rutin were observed in 75:25 ratio of NH_4_^+^:NO_3_^−^, followed by the ratio of 0:100. Moreover, the highest amounts of chlorogenic acid, cinnamic acid and apigenin were detected in the 0:100 ratio of NH_4_^+^:NO_3_^−^. When plants were exposed to higher levels of NH_4_^+^ showed greater amounts of rosmarinic acid and p-Coumaric acid. The plant responses to ammonium are profoundly influenced by the plant nutritional status, with particular regard to the availability of nitrate^74^. High levels of ammonium in the nutrient solution appear to cause stress in plants, and on the other hand stimulate the production of metabolites such as phenolics in Moldavian balm. It has been suggested that PAL activity is increased at high level of NH_4_^+^ and the production of metabolites is induced through the phenylpropanoid pathway^75^. However, there is not further information available concerning the impact of different N forms on accumulation of phenolic compounds. Phenolics are interesting specially due to their antioxidant, anti-inflammatory and anti-diabetic properties^65,66^ . They are considered to be a vital human dietary component and exhibit a tremendous antioxidant activity as well as other health benefits^67^. This research also shows that the providing of ammonium to Moldavian balm plants in DWC system could have lower impact than nitrate on the quantity enhancement of these constituents, indicating that this nutritional strategy could be suitable to improve the technological and nutraceutical value of Moldavian balm.

**Table 3:** Phenolic compounds quantified by HPLC with the calibration curve, correlation coefficient and limits of detection (LOD) and quantification (LOQ).

Phenolic compounds	Retention time	Calibration curve	R^2^	LOD (ppm)	LOQ (ppm)
Gallic acid	4.1	y = 56.859× − 260.94	0.8573	31.08	94.19
Caffeic acid	8.6	y = 52.066× − 41.597	0.9965	4.49	13.60
Chlorogenic acid	9.1	y = 18.482×− 42.177	0.9688	13.68	41.45
Rutin	10.3	y = 127.2×− 61.312	0.9961	4.79	14.50
p-Coumaric acid	10.7	y = 7.6719×− 5.4772	0.9927	6.54	19.81
Quercetin	13.3	y = 18.443×− 8.9102	0.9890	8.03	24.35
Cinnamic acid	14.3	y = 142.04× + 43.786	0.9973	3.96	12.01
Apigenin	14.56	y = 22.82× + 1.7425	0.9976	3.72	11.27

**Table 4:** The phenolic compounds of *D. moldavica* in response to the different NH_4_^+^:NO_3_^−^ ratios.

NH_4_^+^:NO_3_^−^ ratios	Phenolic compounds (mg/100 g)								
	Gallic acid	Caffeic acid	Chlorogenic acid	Rutin	p-Coumaric acid	Rosmarinic acid	Quercetin	Cinnamic acid	Apigenin
100:0*	–	–	–	–	–	–	–	–	–
75:25	7.34 ± 0.05a	14.54 ± 0.07a	3.84 ± 0.05c	7.64 ± 0.03a	2956.46 ± 0.07a	15.86 ± 0.03a	47.62 ± 0.03a	0.84 ± 0.02b	6.34 ± 0.06b
50:50	5.64 ± 0.07c	0.52 ± 0.01c	3.66 ± 0.07d	0.36 ± 0.01d	1328.26 ± 0.04c	2.08 ± 0.02c	1.00 ± 0.02d	0.54 ± 0.03d	2.90 ± 0.03c
25:75	4.24 ± 0.07d	0.44 ± 0.02d	8.40 ± 0.04b	0.72 ± 0.02c	1178.16 ± 0.03d	1.82 ± 0.05d	1.54 ± 0.04c	0.74 ± 0.04c	1.92 ± 0.02d
0:100	5.9 ± 0.04b	1.78 ± 0.03b	21.24 ± 0.06a	2.78 ± 0.03b	2036.48 ± 0.06b	9.44 ± 0.04b	8.82 ± 0.04b	2.42 ± 0.06a	9.54 ± 0.07a

*The plants of 100 NH_4_^+^:0 NO_3_^−^ treatment was destroyed in mid-growth stages, due to ammonium toxicity.

### Antioxidant activity by DPPH and FRAP assays

Natural antioxidants are widely distributed in foods and medicinal plants. The effective extraction and proper assessment of antioxidants from food and medicinal plants are crucial to explore the potential antioxidant sources and promote their application in functional foods, pharmaceuticals and food additives^76^,^77,78^.

**Figure 5:**
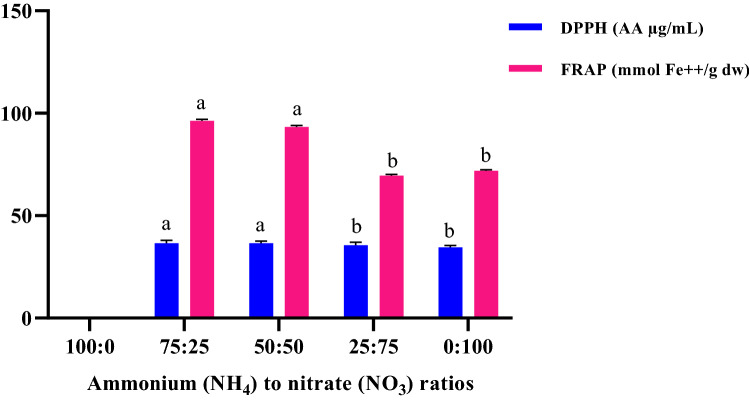
The antioxidant activity in different ratios of NH_4_^+^:NO_3_¯ by DPPH and FRAP assays. *The plants of 100 NH_4_^+^:0 NO_3_^−^ treatment was destroyed in mid-growth stages, due to ammonium toxicity.

The results showed that antioxidant activity by FRAP and DPPH assays in *D. moldavica* was influenced by different ratios of NH_4_^+^:NO_3_^−^ (*p* < 0.01). The highest and lowest antioxidant activity in both DPPH and FRAP methods was obtained in 75:25 and 0:100 ratios of NH_4_^+^:NO_3_^−^, respectively, which are exhibited in Fig. 5. Moldavian palm plants treated with high concentration of ammonium in the nutrient solution, showed significantly a greater amounts of antioxidant activity. The evaluated phenolic compounds (including gallic acid, caffeic acid, p-coumaric acid, rosmarinic acid and quercetin) had strong correlation with FRAP assay than the DPPH method. These compounds most widely used as a donor of electrons and prevents oxidative damages arising from free radicals^79,80^. In addition, results suggested that coumaric acid and quercetin are potent antioxidants with proven therapeutic efficacy^81,82^. Our experiment is also in consistent with other researches (Jakovljević et al.^84^, Okello et al.^83^, Prinsi et al.^31^). On a large scale, high phenolics content are directly related to higher resistance of plants to pests and diseases, and thus a precise regulation of N supplementation could present agroecosystem related advantages by reducing pesticide usage in agriculture. Generally, these natural antioxidants, especially polyphenols and carotenoids, exhibit a wide range of biological effects, such as anti-inflammatory, antibacterial, antiviral, anti-aging, and anti-cancer^77,78^. Considering their beneficial effects for health, the efficient extraction methods of natural antioxidants, appropriate assessment of antioxidant activity as well as their main resources from food and medicinal plants are drawing great attention in food science and nutrition.

### GC and GC-MS analysis of essential oil (EO)

Essential oils are widely used in pharmaceutical, sanitary, cosmetics, agriculture and food industries for their bactericidal, virucidal, fungicidal, anti-parasitical and insecticidal properties^85–87^. The detailed volatile constituents of *D. moldavica* EO were analyzed by GC and GC/MS in different treatments, and fifty-seven compounds were detected (Table 5). The major compounds in *D. moldavica* EO were identified as neral, geranial, geraniol and geranyl acetate. Our results confirms previous studies conducted in Iran, reported that neral, geranial, geraniol and geranyl acetate are the predominant components of Moldavian balm EO^88,89^. The amount of EO components were widely varied by different NH_4_^+^:NO_3_^−^ ratios (Table 5). By applying different ratios of NH_4_^+^:NO_3_^−^, the geranial and geranyl acetate contents in *D. moldavica* EO decreased from 32.76 to 19.89% and from 31.08 to 12.24% respectively, which are presented in Table 5. With different ratios of 75:25, 50:50, 25:75 and 0:100, 21, 14, 18 and 45 compounds were identified, respectively. The highest geranial and geranyl acetate content were obtained in 75:25 ratio of NH_4_^+^:NO_3_^−^, whereas the lowest geranial and geranyl acetate content were obtained in 0:100 ratio of NH_4_^+^:NO_3_^−^. Also, the highest (26.34%) and lowest (4.9%) amounts of geraniol was identified in 25:75 and 75:25 ratios of NH_4_^+^:NO_3_^−^, respectively. The highest (42.40%) content of neral was identified in 50:50 ratio of NH_4_^+^:NO_3_^−^, and the application of 0:100 ratio of NH_4_^+^:NO_3_^−^, resulted in lowest (14.83%) content of neral. In addition, the maximum (87.48%) and minimum (63.85%) levels of total dominant compounds (neral+geranial+geraniol+geranyl acetate) were obtained in 25:75 and 0:100 ratios of NH_4_^+^:NO_3_^−^. The best treatment for major compounds yield was 25:75 ratio of NH_4_^+^:NO_3_^−^. The variations in the volatile constituents of *D. moldavica* EO at different NH_4_^+^:NO_3_^−^ ratios may be due to supply various quantities of nitrate (NO_3_^−^) to the *D. moldavica*. Nitrogen plays a key role in the production of EO in medicinal herbs^90^. N also as an important factor stimulates specific biosynthetic pathways of EOs in medicinal herbs^91^. Nitrogen increases the efficiency of photosynthesis by increasing leaf area, chlorophyll content, and enzymes activity thereby improving the production of EO in medicinal plants^92^. Furthermore, N is as important element in higher plants involved to biosynthesis of many organic structures including amino acids, enzymes, and etc. which are essential for EO production pathway^93^.

**Table 5:** The essential oil compositions of *D. moldavica* in response to the different NH_4_^+^:NO_3_^−^ ratios.

Component	NH_4_^+^:NO_3_^−^ ratios				
	100:0*	75:25	50:50	25:75	0:100
α-pinene	–	0.22	–	–	–
6-Methyl-5-hepten-2-one	–	1.21	2.76	1.94	1.76
3-Methyl-5-methoxy-1-pentanol	–	0.26	–	–	–
Geranyl acetone	–	0.48	–	–	–
2-Butynol	–	0.54	–	–	–
Neral	–	**16.59**	**42.40**	**25.71**	**14.83**
Naphthene	–	0.20	–	–	–
2,6-Octadiene, 4-methyl-	–	1.62	3.46	1.55	–
Geranial	–	**32.76**	**–**	**20.20**	**19.89**
Geraniol	–	**4.90**	**20.80**	**26.34**	**16.89**
Geranyl acetate	–	**31.08**	**22.36**	**15.23**	**12.24**
Geranyl linalool	–	–	–	–	1.58
Farnesyl acetate	–	0.43	0.59	–	0.37
Linalyl formate	–	1.88	–	–	0.30
Geranyl formate	–	3.16	3.09	–	0.78
Nerol	–	1.64	0.67	0.20	–
Nerol acetate	–	–	–	–	0.83
Farnesol	–	1.03	0.32	2.35	5.64
Farnesene	–	–	–	0.46	0.56
β-farnesene	–	0.15	–	0.50	0.58
6,11-Dimethyl-2,6,10-dodecatrien-1-ol	–	0.17	–	0.25	1.89
Gitoxigenin	–	1.30	–	–	–
3-Methylenecycloheptene	–	–	0.14	–	–
Linalool	–	–	1.43	1.68	0.84
Dehydrolinalool	–	–	–	–	0.24
1,6-Dimethylspiro[4.5]decane	–	–	0.23	0.22	–
Spiro[2.5]octane	–	–	1.33	–	–
Vinylcyclooctane	–	–	0.36	0.47	0.30
Vinyl hexanol	–	–	–	0.10	0.32
2-Decyne	–	–	–	1.38	–
Germacrene-D	–	–	–	1.07	1.66
Nerolidol	–	0.13	–	0.21	0.50
5-Decen-1-ol, (E)-	–	–	–	–	0.26
2-Octene, 2-methyl-6-methylene-	–	–	–	–	0.20
Citronellal	–	0.10	–	–	0.30
Vinylcyclohexane	–	–	–	–	3.03
Rosefuran epoxide	–	–	–	–	0.31
Myristoleyl alcohol	–	–	–	–	0.20
Pulegone	–	–	–	–	0.32
Geranic acid	–	–	–	–	2.38
α -cubebene	–	–	–	–	0.52
Caryophyllene	–	–	–	–	1.09
Caryophyllenol II	–	–	–	–	0.40
α- Caryophyllene	–	–	–	–	0.82
Caryophyllene oxide	–	–	–	–	0.94
β-ionone	–	–	–	–	0.24
γ-cadinene	–	–	–	–	0.22
Δ-cadinene	–	–	–	–	0.40
Copaene	–	–	–	–	0.43
Fitone	–	–	–	–	0.24
Phytol	–	–	–	–	2.49
α -damascone	–	–	–	–	0.10
2,5-Dimethylhex-5-en-3-yn-2-ol	–	–	–	–	0.19
1,6-Dibromohexane	–	–	–	–	0.29
Tricosane	–	–	–	–	0.15
β -bisabolene	–	–	–	–	0.13
p-heptylacetophenone	–	–	–	–	1.81
Total amount of compounds		99.85	99.94	99.86	99.46

*The plants of 100 NH_4_^+^:0 NO_3_^−^ treatment was destroyed in mid-growth stages, due to ammonium toxicity.

Significant values are in [bold].

## Conclusion

Crop production in hydroponic systems take many advantages compared to open fields. Higher yield, good quality and shorter growing periods are some benefits of hydroponically plant growing. Since the roots are constantly supplied with oxygen-rich nutrient solution in DWC, the plants grow very quickly and vigorously. Along with the aboveground parts, plant roots obtained from this system are not spoiled by substrate particles and can be easily removed from the nutrient solution without any damage or lose. This is important especially when such plants with valuable roots should be grown. Nitrogen is the most important macro nutrient in plant nutrition that have many roles in plant. In addition to nitrogen concentration in nutrient solution, the available forms of nitrogen and their ratio have significant effects on growth, photosynthesis, yield and plant quality. The two major forms of nitrogen normally used by higher plants are nitrate and ammonium. There is not further information available concerning the impact of different N forms on Moldavian balm. The present study was done to determine the effect of different ammonium to nitrate ratios on morphophysiological, nutrient contents, phenolic compounds, essential oil components, and antioxidant capacity of *D. moldavica* in DWC system. The results revealed that studied traits of *D. moldavica* were considerably influenced by the different ratios of NH_4_^+^:NO_3_^−^. The NH_4_^+^ as sole N source showed toxic effects being visible on growth and morphological traits. The supply of NO_3_^−^ as N source increased the accumulation of some phytochemicals (TPC, TFC, and TCC) in plant tissues. For most traits, a mixture of nitrate and ammonium is recommended as an excellent fertilizer rather than just nitrate or ammonium. These results suggest that the management of N source could contribute to improve morphophysiological traits, antioxidant activity, and phytochemical compounds in Moldavian balm.

## Data Availability

The datasets used and/or analyzed during the current study available from the corresponding author on reasonable request.
